# Supplementary material for: “Let’s see what happens:”—Women’s experiences of open-label placebo treatment for menopausal hot flushes in a randomized controlled trial
Source: PLoS One. 2022 Nov 4;17(11):e0276499. doi: 10.1371/journal.pone.0276499 (PMC9635716; doi:10.1371/journal.pone.0276499)
Supplement: S1 Appendix — (DOCX) [file pone.0276499.s001.docx]

**S1 Appendix.** Placebo rationale

1. The placebo effect is powerful. Patients reported symptom improvement after taking placebos in double-blind drug trials, including hot flush trials. However, these participants were unaware whether they received a placebo or medicine, which differed from this trial. A few studies have shown that placebos without deception can have beneficial effects.

Just like when we take a medication, it was shown that taking a placebo can also lead to measurable changes in the body and the brain. Thus, the placebo effect is “real”.

1. The body may react to the pill intake automatically (Depending on the patient’s prior knowledge, an example is given, e.g., the Pavlov dog or food poisoning for which certain foods cause queasiness and nausea).
2. Believing in a positive effect can help but is not necessary for the placebo to be effective. In other words, even if you are skeptical or do not believe in it at all, the placebo might work anyhow.
3. Taking the pills faithfully twice a day is crucial since the custom of pill intake can contribute to the effect.

Also, adhering to the instructions is vital for the validity of the results. If one woman only took a couple of pills but were allocated to the placebo group, she would be more similar to a participant in the comparison group. Comparing the placebo and the comparison groups would be less credible.

1. We do not know whether placebos without deception can reduce hot flushes. Therefore, in case you are allocated to the placebo group next week, I would like to encourage you to give it a try and see what happens.
