# Supplementary material for: “Let’s see what happens:”—Women’s experiences of open-label placebo treatment for menopausal hot flushes in a randomized controlled trial
Source: PLoS One. 2022 Nov 4;17(11):e0276499. doi: 10.1371/journal.pone.0276499 (PMC9635716; doi:10.1371/journal.pone.0276499)
Supplement: S2 Appendix — (DOCX) [file pone.0276499.s002.docx]

**S2 Appendix.** Research team description

The interviewer MLF (female medical student) was unknown to most participants, except for one patient who met the interviewer as the assessor in the RCT (i.e., administering the questionnaires and explaining how to complete the diary). This study was her first involvement in placebo research. YP is a Ph.D. psychologist and associate researcher in the field of placebo studies. She accompanied all participants as the clinician, i.e., informed them about the study and the placebo treatment, handed out the pills, and conducted the follow-up sessions inquiring about potential adverse events. TJK is a leading researcher in the field of placebo effects and has conducted the first RCT on OLP effects. Currently, he is the director of the Harvard-wide Program in Placebo Studies and the Therapeutic Encounter at the Beth Deaconess Medical Center. TJK has long-term expertise in qualitative and quantitative research. YN is a clinical psychologist trained in cognitive-behavioral methods and a professor of clinical psychology. She researches expectations, placebo- and nocebo effects, and side effects of psychotherapy. None of the researchers underwent menopausal transition themselves.
