# Supplementary material for: “Let’s see what happens:”—Women’s experiences of open-label placebo treatment for menopausal hot flushes in a randomized controlled trial
Source: PLoS One. 2022 Nov 4;17(11):e0276499. doi: 10.1371/journal.pone.0276499 (PMC9635716; doi:10.1371/journal.pone.0276499)
Supplement: S3 Appendix — (DOCX) [file pone.0276499.s003.docx]

**S3 Appendix. Original quotations in German (with context information and English translation)**

Quotations are listed in same order as in the results. The parts of the quotation displayed in the main body of text is displayed in italic. All contextual information is displayed in regular font. If there was no immediate question before the quotation, the first column (interviewer question) was left empty. All names were changed.

**Cluster A, Theme (1) Openness: Low expectations and hope**

| **Qt. #** | **Interviewer question** | **Participant statements** | **Participant** |
| --- | --- | --- | --- |
| 1 |  | Aber *konkret erhofft hab‘ ich mir nichts, sondern ich hab‘ gedacht och, probierst du mal aus. So mit Offenheit und...ja Neugier bin ich daran gegangen.*  [*I didn’t expect anything in particular, but instead I thought, well, just give it a try. I approached it with some kind of openness and … yes, curiosity.*] | Marlene |
| 2 | Okay, schön. Haben Sie denn eine Idee für sich selbst warum das Placebo so gut gewirkt hat?  [Okay, great. Do you have any idea for yourself why the placebo worked so well?] | *Vielleicht weil – ich bin ganz entspannt da rein gegangen, also ich hab‘ mir vielleicht gar nicht so große Erwartungen hoch gesetzt. Oh, das muss jetzt unbedingt klappen. Ich hab‘ jetzt diese… Erwartungen. Sondern ich* bin ganz entspannt reingegangen und *hab gedacht: Mensch, entweder klappt es oder es klappt auch nicht. Und wenn es nicht klappt, dann sei nicht enttäuscht, irgendwie halt.*  [*Maybe because - I went in quite relaxed, so maybe I didn't have such high expectations. Oh, it has to work out now. I now have these … expectations. Instead I* went in relaxed and *thought: Gosh, either it works or it doesn’t work. And if it doesn’t work, then don’t be disappointed, something like that.*] | Andrea |
| 3 | Was mich nochmal interessiert im Vergleich zu dem, was Sie erhofft oder sich gewünscht haben, wenn Sie das unterscheiden können ähm..was waren Ihre Erwartungen?  [What I am still curious about is that compared to what you hoped or wished for – if you can distinguish it- ehm… what were your expectations?] | *Also natürlich hab ich mir einen Erfolg erhofft, aber meine Erwartungen waren glaub ich anfangs nicht so...nicht so..groß.*  [*Well of course I was hoping for a success, but my expectations in the beginning I think weren’t, well… not so…not so high.*] | Ingrid |
| 4 | Und was hatten Sie sich von der Placebo Behandlung erwartet?  [And what did you expect from the placebo treatment?] | Naja, schon, dass die weniger werden, ne? Ich hab, naja..nee, erwartet nicht. *Erwartet hab‘ ich gar nichts. Gehofft hab’ ich das. Aber ich hab’ auch ein bisschen gedacht, naja gut, ist ja ein Placebo, ne? Was soll da schon groß passieren? (lacht)*  [Well, that they’re getting fewer, right? I expected, well…no, not expected. *I didn‘t expect anything. I was hoping. But I also thought, well, it‘s a placebo, right? What could possibly happen there after all? (laughs)*] | Gabriele |
| 5 | Und was hatten Sie erwartet?  [And what did you expect?] | […] *Ich bin da ziemlich,* was man so oberflächlich nennen kann, unbewusst, *unbedacht rangegangen.* *Also ähm...auch, dass es mir was bringt, klar, zumindest es ein bisschen abmildert ähm…aber jetzt nicht so die große Erwartung.* […] *es wär für mich auch nicht logisch gewesen, dass ich äh "geheilt" rausgehe da aus der Sache.*  [[…] *I approached it what* you can superficially call unconsciously, *without too much thinking. Well, ehm also that it’s gonna help me, sure, at least that they [the hot flushes] are a bit reduced, but - not such high expectations. […] It also wouldn’t seem logical to me, that -ehm - I’d come out “all healed.”*] | Edith |
| 6 | Was haben Sie sich zu dem Zeitpunkt erhofft, wissen Sie das noch? Vor Beginn der Studie?  [What were you hoping for at the time, do you remember? Before the start of the study?] | […] *Also ich hatte die Hoffnung tatsächlich, dass diese, dass die Hitzewallungen gehen, aber ich war eigentlich neutral eingestellt.* Ich wusste jetzt nicht, funktioniert des, oder funktioniert des nich', aber schon so positiv, dass ich dachte, es könnte funktionieren (Interviewer: Hm hm). Ich sag mal, ich war so neutral eingestellt, *ich hab' nicht gedacht das hilft oder das hilft nicht, sondern - ich lass es auf mich zukommen.*  [[…] *Well, in fact, I had hopes that these hot flushes would disappear, but my attitudes were technically neutral.* I didn’t know, does it work, or does it not work, but still positive enough that I thought, it could work out. (Interviewer: hm hm). *I didn’t think, it will or won’t help, but rather – Let’s see what happens.”*] | Karin |
| 7 | Hatten Sie da bisher irgendwelche anderen Hilfsangebote noch oder wie hat sich bei Ihnen die Suche jetzt gestaltet über so einen längeren Zeitraum? Haben Sie aktiv gesucht, oder…?  [Did you have any other service options or how did the search look like over such a long period of time? Did you look actively, or…?] | […] *Dachte ich Mensch, versuch es doch mal, jetzt nochmal, vielleicht so das letzte mal, vielleicht klappt das. Und wenn nicht dann hätte ich, irgendwie halt, dann gar nichts mehr gemacht. […] Ja weil ich hab dann irgendwie halt gedacht, Mensch, ah ok, dann bin ich eben halt der eine von ganz vielen wo, es eben halt nichts gibt, was hilft.*  [[…] *I thought, gosh, maybe you should give it a try, one more time, maybe for the last time, maybe it’ll work. And if not then I would have – I guess – not done anything anymore [...]. Yes because then I somehow thought, gosh, well ok, then I’m probably one of those many women for which nothing works.”*] | Andrea |
| 9 | Was haben Sie sich zu dem Zeitpunkt erhofft?  [What were you hoping for at that point in time?] | *Also sag ich mal bewusst..unbewusst als, schon eher, dass ich äh..ähm...Hilfe rufend, […] dann hab ich gedacht aah, hoffentlich bekomm ich die Placebo, also..war es wieder dieses ja hoffentlich, hoffentlich* hilft mir das, bringt mir das was...*erleichtert es mir die Hitzewallungen.*  [*Well let’s just say consciously- unconsciously rather – ehm - I was crying for help. […] then I thought, ahhh, hopefully I’ll receive the placebos, yeah, there it was again, this -hopefully, hopefully* it’s gonna help, it’s gonna do something…*will alleviate the hot flushes*] | Edith |
| 10 | Was hat Sie zu der Studienteilnahme bewegt?  [What motivated you to participate in the study?] | […] So…*ich bin jetzt tatsächlich so weit zu überlegen mache ich ne Hormontherapie* und und und äh, *weil ich schlaf nicht mehr, so. Ich schlaf verdammt nochmal nicht mehr und ehm..es geht so nicht mehr.*  [[…] Well.. *I was actually going that far of thinking whether I should start with hormone therapy* and so and so and so on ehm, *because I just don’t sleep anymore, period. I don’t fricking sleep anymore and – ehm- this can’t go on like this.*] | Meike |
| 11 |  | *Weil man sich eben auf ein komplett neues Feld bewegt und daran gekoppelt ist ja auch eine große Hoffnung*, also eine große Hoffnung, dass es vielleicht tatsächlich irgendwann ne Möglichkeit und ne Weg gibt diese gesamte Problematik oder diese gesamte Situation halt irgendwie angehen zu können, *also das schwingt auch irgendwie mit rein, also eher so was Großes im Hinterkopf.*  [*Because you’re moving into a completely new field and there’s also a lot of hope attached to it,* like a big hope that there is maybe really going to be a possibility or a way to address this whole problem or this whole situation somehow*, so somehow something as big as that is also in the back of your head.*] | Meike |
| 12 |  | *Deswegen haben die vielleicht bei mir auch so gut angeschlagen, ich weiß es nicht. Weil ich vielleicht nicht mich so verkrampft hab oder ganz entspannt reingegangen bin.*  [*This is why maybe they worked so well for me, I don’t know. Because maybe I did not feel as tense and went in completely relaxed*] | Andrea |
| 13 | Haben Sie denn eine Idee warum das Placebo Ihnen geholfen hat?  [Do you have any idea why the placebo helped you?] | *Wenn ich jetzt negativ oder so, mit einer negativen Einstellung daran gegangen bin, rangegangen wäre, äh bin ich der Meinung, also hätte es mir vielleicht nicht geholfen. Oder wenn ich versucht hätte das zu kontrollieren.*  [*If I was negative or something – approached it with a negative attitude – ehm- then I think it probably wouldn’t have helped me. Or if I tried to control it.*] | Edith |

**Cluster A, Theme (2) Curiosity**

| **Qt. #** | **Interviewer question** | **Participant statements** | **Participant** |
| --- | --- | --- | --- |
| 1 | Gab es da irgendetwas bestimmtes, was Sie an dem Aushang oder Flyer, den wir ja hatten, angesprochen hat, sie Sie das das erste mal gesehen haben?  [Was there anything in particular about the poster or flyer of which we talked about that appealed to you when you first saw it?] | *Ja, ich fand das ganz extrem… interessant, dass es eben halt ja nur Traubenzucker ist.*  [*Yes, I found that extremely… interesting, that it really is just sugar.*] | Andrea |
| 2 |  | Es ist ein total spannendes Feld, weil man sichs nicht erklären kann und *Sachen, die man sich nicht erklären kann, machen ja neugierig, ne?*  [It’s a super fascinating field, because you can’t explain it and *things that you can’t explain make you curious, right?*] | Meike |
| 3 | (Prompt) Haben Sie sich da nochmal zusätzlich informiert oder viel mit Angehörigen drüber gesprochen...  [Have you done any additional research on this or talked much about it with family members...] | *Ich hab das allen möglichen Leuten erzählt, auf jeden Fall, weil ich das einfach total interessant fand.*  [*I told all kinds of people about it – for sure, because I just found it super interesting.*] | Gabriele |
| 4 | Und wenn Sie das nochmal vergleichen mit den Schüßler-Salzen mit Erwartung und Hoffnung, weil sie grad gesagt haben dann sind die Erwartungen bei den Salzen vielleicht nicht so hoch...gabs da denn einen großen Unterschied zu dem Placebo?  [And if you’d compare it again with the homeopathic salts considering expectations and hope, because you just said that then the expectations for the salts were probably not as high…was there a big difference compared to the placebo?] | *[…] Ehm..also ich glaube bei den Schüßler-Salzen war die Erwartung,* also Erwartung ist für mich eher emotionsloser, also Hoffnung ist einfach sehr an Emotionen gekoppelt ehm..und die Erwartungen sind eher rationaler Natur und es klappt oder es klappt nicht und wenns nicht klappt, ja, war nen Versuch wert, peng. Hoffnung ist da nicht, *weil ich hab bei Schüßler-Salzen ja bereits die Erfahrung gemacht, dass das wirken kann*, so […] *Also wenn ich Schüßler-Salze nehme, dann bin ich nicht neugierig.*  [*Hm well I think for the homeopathic salts there were expectations*, so expectations are more emotionless for me, so, hope is just very much attached to emotions ehm.. and the expectations are rather of rational nature and it works or it doesn’t and if it doesn’t, well, still worth a try, peng. There is no hope there *because I’ve already made the experience that homeopathic salts can work I...]. And when I take homeopathic salts, I’m not curious.*] | Meike |
| 5 | Und wenn Sie das vergleichen mit dem Placebo in der Studie bei uns mit den Erwartungen damals zur Traubensilberkerze, wie würden Sie da [sagen]?  [And if you compare it – the placebo in our study and your expectations of the black cohosh back then, what would you [say]?] | […] *Aber Erwartungen waren auf jeden Fall höher und das macht es ja noch faszinierender, dass das hier..ja, funktioniert hat. Das ist schon echt..ja...klasse.*  [[…] *The expectations were definitely higher, and that’s what makes it even more fascinating, that this has worked. That was really…well, cool.*] | Gabriele |
| 6 | Und Sie hätte das Placebo, wenn ich das richtig verstanden hab, ganz gerne noch weiter genommen? Was glauben Sie hätte das geändert?  [And you would have liked to continue taking the placebo, if I understand correctly? What do you think would have changed?] | Wär vielleicht noch besser geworden (lacht). So, hm? Äh..ja. *Ich hätte gedacht so naja, es kann ja noch mehr Auswirkungen haben, so vielleicht, wenn man länger das nimmt.*  [Maybe would have gotten even better (laughs). Like that right? Ehm yeah. *I would have thought like - the placebo would have even more effects, something like that, if you took it longer*] | Eva |

**Cluster A, Theme (3) Placebos: Only potential gains, zero losses**

| **Qt. #** | **Interviewer question** | **Participant statements** | **Participant** |
| --- | --- | --- | --- |
| 1 |  | *Weil das ist ja wirklich...ja, schon toll, ne? Dass das ohne körperliche Belastungen durch irgendwelche Stoffe eine Verbesserung erzielt wird. Das ist ja perfekt im Grunde.*  [*Well that’s really, I mean great, right? That you can obtain improvements without burdening the body with any substances. That’s actually perfect.*] | Gabriele |
| 2 | Und haben Sie selbst eine Idee warum das Placebo Ihnen geholfen hat?  [And do you yourself have an idea why the placebo helped you?] | *Joa, ich glaub weils einfach, weil ich sowieso darüber positiv gedacht hab als jetzt zu denken, oh nee, ist ja nur ein Placebo…glaub ich.*  [*Well, I think it’s just because I have thought about it [the placebos] positively instead of thinking, gosh no, it’s just a placebo… I think.*] | Eva |
| 3 |  | *Ich glaub nicht an Homöopathie und Schüssler-Salze, also, ich glaub auch, dass es eher ein Placebo-Effekt ist (lacht). Ich glaub aber an den Placebo-Effekt!*  [*I don’t believe in homeopathy and homeopathic salts, I think that that’s also rather a placebo effect (laughs). But I do believe in the placebo effect!*] | Karin |
| 4 |  | *Hitzewallung ist ja nun keine Krankheit,* sondern..*und das muss man auch nicht gleich mit Keulen bearbeiten* sondern halt..eher, ja das hört sich immer so pathetisch an, also mit Einklang der Natur (ironisch), *aber vielleicht einfach ein bisschen natürlicher behandelt und nicht ignoriert, so. Das würde ich mir wünschen, wenn da so ein bisschen mehr ankommt.*  [*Hot flushes are not a disease,* but instead.. *and you don’t have to immediately get the [chemical] clubs out,* but rather..well, it always sounds so dramatic, well, in harmony with nature (ironical), *but rather treating it a bit more naturally and not just ignoring it, yeah. This is something that I'd hope for, that this comes through more.]* | Edith |

**Cluster A, Theme (4) Lack of explanation and fascination for placebo effects go together**

| **Qt. #** | **Interviewer question** | **Participant statements** | **Participant** |
| --- | --- | --- | --- |
| 1 | Da die anderen Sachen wie Mönchspfeffer, Traubensilberkerze oder auch ihre homöopathischen, was auch immer das war, ja nicht gewirkt hatten und Sie selbst eben gesagt haben, dass Sie so überrascht waren, haben Sie eine Idee warum das bei dem Placebo dann anders war?  [Since the other things like monk’s pepper, black cohosh and also the homeopathic ones, whatever those were, did not work and like you yourself just said, you were surprised, do you have an idea of why it was different with the placebo?] | Also Frau Pan hat das ja so ein bisschen so erklärt, dass *der Körper das einfach ein bisschen anders sieht als der Kopf*, so. Also..sie hatte das mit einer Lebensmittelvergiftung verglichen, dass man wenn man dann das Lebensmittel, wo man sich vergiftet hatte das sieht und man weiß das ist aber jetzt gut, der Körper trotzdem, einem trotzdem übel ist und man kann es nicht essen. *Und,* dass *der Körper einfach weiß Tabletten sind gut für mich, auch wenn der Kopf sagt da ist ja nichts drin, so, keine Ahnung. Es klingt für mich ein bisschen einleuchtend und da mach ich mir auch gar nicht weiter Gedanken drüber.*  [*Well* Ms Pan did explain it a bit like that that *the body sees it a bit differently than the head* Well…she compared it to food poisoning, that if you looked at the food with which you poisoned yourself and you know now this time it’s fine that the body, nonetheless, that one still gets nauseous and you can’t eat it. *And that the body knows that pills are good for me, even if the head says there’s nothing in there, something along those lines, I don’t know. And that kind of made sense to me, so I won’t think about it any further.]* | Gabriele |
| 2 |  | ja, *nach dem ich dem die abgesetzt hatte, sagte – wo es dann wieder etwas stärker wurde- sagte mein Mann, ach Mensch da hättest doch weiter die Placebos nehmen sollen (lacht)!*  [yes, *After I discontinued the pills, and when they [the hot flushes] have gotten more severe again, my husband said – Oh dear, you should have kept taking those placebos! (laughs)*] | Karin |
| 3 | Und wenn Sie das in Ihren eigenen Worten einmal jemandem erklären müssten, was würden Sie sagen ist Placebo?  [And if you had to explain this in your own words to someone, what would you say is a placebo?] | *Also ich hab ja auch ähm im Vorwege hab ich ja auch meiner Familie zum Beispiel erzählt, dass ich teilnehme und hab gesagt: gibt zwei Gruppen, die einen kriegen nix und die anderen kriegen Tabletten mit nix, so. Das war das und das, finde ich, erklärt das ganz gut, also…und das macht es aber auch, finde ich, deutlich...wie merkwürdig das eigentlich ist. (lacht) Ja.*  [*Well I also – ehm- had told my family beforehand that I was taking part, and I said: there are two groups, one group receives nothing, and the other group receives pills with nothing, like that. That’s just what it was, and I think that’s a good explanation…But that does show – I think quite clearly – how weird this all is (laughs), yes.*] | Gabriele |
| 4 | Und haben Sie selbst eine Idee warum das Placebo bei Ihnen gut gewirkt hat?  [And do you yourself have an idea why the placebo worked well for you?] | *Ich weiß es läuft halt einfach super viel ab,* irgendwie sowas, *von dem wir nicht wissen,* also was einfach unter unserem Radar abläuft und ehm..und was nicht mal unterbewusst abläuft, *sondern was auf Körperebene halt irgendwie abläuft* […] *der Mensch meint er kriegt das irgendwie hin immer zu begreifen, was irgendwo abgeht.* Vielleicht ists auch besser, er tuts halt irgendwie nicht, ne? Wer weiß das schon? Ehm..so und..ja, das ist einfach, keine Ahnung, also das ist ganz viel nicht wissen ehm...*aber ich glaube aber an viele Sachen und ehm..könnte mir vorstellen, dass das auch mit eine Rolle spielt, weil ich halt sehr offen für Unbenanntes.*  [*I know that there are tons of things,* something like that, *that we don’t understand yet,* like what’s just below our radar and ehm..and is not even happening unconsciously, *but rather it’s happening just on a physical level somehow […] The human being supposes that he can always understand what’s going on.* Maybe it’s also better that he kind of doesn’t, you know? Who knows? Ehm..so and…yes, that’s just, I don’t know, well that’s a lot of not knowing ehm.. *But I believe in many things, and I could imagine that this played a role too, as I’m just very open for what’s unlabeled.*] | Meike |

**Cluster B, Theme (5) Seeking evidence for improvement**

| **Qt. #** | **Interviewer question** | **Participant statements** | **Participant** |
| --- | --- | --- | --- |
| 1 |  | *Ich hab jetzt nachts auch nicht mehr so diese großen Hitzewallungen, so dass ich mich neues Oberteil anziehen muss.*  [*Also, at night I don’t have those big hot flushes anymore, for which I would have to wear a different shirt.*] | Andrea |
| 2 |  | *Ich hatte* immer Stufe, also *vermehrt Stufe eins und vermindert Stufe drei*, ja genau*.*  [*I had […] more level one and less level three, yes.*] | Edith |
| 3 | Und hat sich neben dem Protokollieren in dem Tagebuch, was Sie ja auch sehr ausführlich eben gemacht haben, sonst noch was an der Wahrnehmung der Hitzewallungen geändert?  [And besides the logging in the diary, which you have done in great detail, has anything else changed in your perception of the hot flushes?] | *Also es ist wirklich deutlich weniger dann auch geworden*, *also vorher, vor der Studie war eben halt extrem gewesen und dann nachher mit den Tabletten oder eben halt auch als ich die vier Wochen keine genommen hab, also es war deutlich dann nachher viel besser.*  [*Well, it really was much less […]. And previously, before the study [it] was also extreme and then later with the pills and also during the four weeks in which I didn’t take any, well that was clearly much better.*] | Andrea |
| 4 | Sie hatten eben schon ein bisschen angefangen zu schildern wie sich die Wahrnehmung der Hitzewallungen geändert hat oder Sie besser unterscheiden konnten aufgrund des Tagebuchführens, wenn die Hitzewallungen während der Studie für Sie anders waren, können Sie das nochmal ein bisschen beschreiben inwiefern?  [You had just started to describe a bit about how your perception of the hot flushes changed or how you could better distinguish due to the protocolling when the flushes were different during the study. Could you describe a bit to me again how?] | *[…] Auch mit der Stimmung, die ja damit auch, ne, also Wechseljahren hat man ja bestimmte Stimmungen, also das ganze Drumherum war ähm..in abgemilderter Form, auf jeden Fall.* Also es war nicht weg, klar, das ist ja auch schwierig. Das äh..ist ja auch ein Teil des Prozesses, aber ähm..*das Herzrasen war weniger, die Panikanfälle,* die ich mitunter hatte, *die waren weniger..oder gar nicht, also wirklich sehr vermindert.*  [*Even the mood, which also is – I mean during menopause you have certain moods, well, all that stuff around it were -ehm- less, definitely.* Well it was not gone, sure, that was also difficult to do. That ehm…that’s also part of the process, but ehm… *Less heart racing, fewer panic attacks,* that I also had, *or not at all, so it was really very diminished.*] | Edith |
| 5 |  | *Wenn ich* *irgendwie die Leistung vielleicht nicht bringen kann oder wenn ich morgens erst um zehn zur Arbeit komme, weil ich die ganze Nacht nicht gepennt hab äh, weil ich Herzrasen hab oder so. Oder, ich mein das hab ich nicht mehr, ne? Aber…das ist tatsächlich weg. Ich hab halt nur Hitzewallungen im Moment wieder mal, so. Und ähm..und auch nicht mehr dieses depressive verstimmt sein sozusagen, das hab ich grad auch nicht.*  [*When I somehow couldn’t effectively perform, or I would only come to work at 10am because I couldn’t sleep all night, uhmm, because I had heart racing or something like that. I mean, I don’t have that anymore, right? That’s actually gone. I only have hot flushes – yet again - right now, yeah. And those depressed moods, I don’t have them either at the moment.*] | Eva |

**Cluster C, Theme (6) Attributions in favor of the treatment**

| **Qt. #** | **Interviewer question** | **Participant statements** | **Participant** |
| --- | --- | --- | --- |
| 1 | Ab wann hatten Sie denn das Gefühl, dass das Placebo bei Ihnen angeschlagen hat?  [At what point were you under the impression that the placebo had started working?] | Mhm…eigentlich ging das, glaub ich, relativ flott los. *Und dann gab es immer mal so ein paar Tage, wo das wieder etwas anstieg*, aber so..ich hab das Gefühl gehabt, dass das gleich mit einer leichten, mit einem leichten Rückgang, also noch in der ersten Woche, nach ein paar Tagen war das schon spürbar. Und...joa, *dann..hab ich so das Gefühl gehabt, dass das mehr so das Essverhalten, wie ich geschlafen hatte und wie halt vielleicht tatsächlich noch externe Stressfaktoren* mich beeinflusst haben, *dass das dann dazu geführt hat, dass wenn es wieder mal so ein, zwei Hitzewallungen mehr waren, so.* *Und das lag nicht daran, dass das Mittel nicht gewirkt hat, sondern das lag halt an dem Druck, der..aus anderen Quellen halt auf mich eingegangen ist.*  [Mhm…to be honest that went off, I think, rather quickly. *There were those couple days in which it increased a bit* but like...I had the feeling that it started right away with a slight, with a slight decrease, so still in the first week, after a couple days it was already noticeable. And...yeah, *then I had the feeling that it’s more my eating behavior and how I have slept and actually maybe external stressors on top* that have influenced me *– that these have caused one or two more hot flushes. And it’s not because that the remedy didn’t work, instead it’s… those strains from other sources that affected me.*] | Marlene |
| 2 |  | *Ja, bei mir haben die Placebos auch geholfen. Ich weiß natürlich nicht‘ob' s an den Placebos lag oder ob es daran lag, dass vorher ja Sommer war und es sehr heiß war* (Interviewer: Hm), da hatte ich die ja sehr stark und *als ich mit der Studie angefangen habe wars Herbst – dann wars halt nicht mehr so warm.*  [*Yes, the placebos also worked for me. Of course, I don’t know whether it’s the placebo and whether it is because that earlier it was summer and very hot* (Interview: hm), there I had them very strongly and *when I initiated the study, it was fall – and not so warm then.*] | Karin |

**Cluster C, Theme (7) Monitoring symptoms helped**

| **Qt. #** | **Interviewer question** | **Participant statements** | **Participant** |
| --- | --- | --- | --- |
| 1 | Das heißt das Protokollieren der Hitzewallungen, was würden Sie sagen, wie das für Sie war?  [The protocoling of the hot flushes how would you say how was it for you?] | […] Also auch hilfreich in der Hinsicht, dass ich vorher, *ich hatte es zwar oft, aber ich wusste trotzdem nicht wie viele das waren, ich hab die ja nicht gezählt. Und das zu sehen, dass das wirklich zehn bis zwölf waren am Tag, das war auch schonmal echt beeindruckend zu sehen, dass ich gedacht hab ja, stimmte. Stimmt das Gefühl, dass es ständig war.*  [[…] and also helpful in that way that, before, *although I had it often, but still I didn’t know how many these were- I mean I didn’t count them. And to see that I actually did have 10 to 12 a day, that was also impressive to see, and I thought – yeah, that was correct- the feeling that I have them all the time was correct.*] | Gabriele |
| 2 | Und weil Sie diese Einstellung, dieses Annehmen schon jetzt ein paar Mal erwähnt haben, wie hat sich, wenn sie sich verändert hat, die Wahrnehmung der Hitzewallungen während der Studie für Sie verändert?  [And because you’ve mentioned this attitude, this acceptance multiple times now, how did, if it did, the perception of the hot flushes change for you?] | *Also ich hätte mich vorher, hätte ich trotzdem meine Hitzewallungen schon als stark eingeschätzt, so rein subjektiv.* […] *Also ich konnte mich da irgendwie ähm…besser einordnen* beziehungsweise tatsächlich musste auch dann feststellen, dass ich ähm...gar nicht so, also was (unverständlich), klar betroffen bin, aaber (langgezogen, lacht) *ich hab das einfach im Laufe der Studie nicht mehr so... einschränkend,* also das war ja auch immer so eine der Fragen, glaube ich, ne? Also wie schränkt mich das *in meinem Alltag* oder in meinem Leben ein oder ja..*auf jeden Fall,* *die Wahrnehmung hat sich zu deutlich...deutlich reduziert. Das hab ich dann nicht mehr so einschränkend oder belastend empfunden.*  [*Well before I thought my hot flushes were severe – purely subjectively spoken.* […] *Well, I could somehow classify myself – ehm- better* or in fact I had to realize that I am ehm… not as much, well what (inaudible), affected, buuut (stretched, laughs), *during the course of the study, I simply didn’t feel as …restricted,* well that was also always one of the questions, I think, right? In terms of how it limits me *in my day-to-day* or in my life or yes…, *definitely, this perception has clearly …clearly changed. Then I no longer found them as limiting or burdensome.*] | Ingrid |
| 3 | Haben Sie während der Studie oder während der Placebo-Einnahme die Hitzewallungen dann auch anders wahrgenommen?  [During the study or during the placebo intake, did you perceive the hot flushes differently?] | *Es ist ja so ein Hand in Hand, also ich bekomm ne Bestätigung, dass es nen bissl besser wird, dann hab ich die Hoffnung, dass das so ist, das bestätigt sich wieder und ehm…mildert dann vielleicht einfach meine Erwartung einer schlechten Nacht etwas.*  [*It goes hand in hand. Well, I receive the validation that it’s gotten a little better, and then I’m hopeful that it is the case, which gets validated again, and -ehm- maybe just attenuates my expectations of having a bad night a bit.*] | Meike |
| 4 | Wie war das für Sie auf einmal drauf achten zu müssen, wo Sie sonst eher als Strategie das Ignorieren angewendet haben?  [How was it for you to, all of the sudden, having to pay attention to it, where you’ve otherwise rather used the strategy of ignoring it?] | Also dadurch, dass sie ja weniger wurden, war das nicht schlimm. Im Gegenteil, *wenn ich dann gesehen hab nur so drei oder vier, das war dann schon gut (strahlt) das so, nochmal so zu sehen, dass es eben echt wirklich weniger geworden ist.*  [Well as they were getting less, it wasn’t so bad. Quite the opposite, w*hen I saw that these were just three or four – well that was really good (beaming) to see that it’s actually really gotten less.*] | Gabriele |
| 5 | Hat sich unabhängig von dem Protokollieren noch irgendwas geändert an der Wahrnehmung?  [Did anything else change about your perception that was unrelated to the protocoling?] | Ja, vielleicht so das negative Gefühl so: oh Gott, jetzt ist eine Hitzewallung. Das ist gewichen einem neutralen Gefühl, weil man sich dann ja selbst beobachtet, also *man ist sozusagen von einem Leidenden zu einem Statistikführenden im Prinzip geworden*. Und das hilft das zu ähm…wie soll ich sagen, neutralisieren und objektivieren, also das ist einfach ein Schritt zurück. Das ist ähm...ja. Es versachlicht das Ganze total.  [Yes, maybe some kind of a negative feeling like: oh jeez, now there is a hot flush. That’s substituted by a neutral feeling, because you observe yourself, so *one has technically transitioned from being the sufferer to the bookkeeper.* And that helps to ehm..how should I put it, neutralize and objectify it, well it’s just a step back. That’s ehm, yeah. It totally factualizes the whole thing.] | Marlene |
| 6 |  | Ehm..und das macht wahrscheinlich auch was mit einem, so..*also..allein schon* deshalb vielleicht, *weil man sich auch im Austausch damit wisschenschaftlich* *sich nen bissl damit* *auseinandersetzen kann* und *gewinnt* dadurch halt ein etwas..also *eine andere Perspektive darauf und dadurch vielleicht auch noch eine gewisse Distanz* so von einem bestimmten Punkt aus *und ist dem nicht so völlig ausgeliefert.*  [Ehm and that’s also doing something with you, *The fact alone* maybe also *that you can explore it a bit scientifically through an exchange, you gain* by that somehow *a different perspective on it and maybe by that, a certain distance,* from a different point, *so you’re not completely at its mercy.*] | Meike |

**Cluster C, Theme (8) Recognizing the influence of psychosocial factors**

| **Qt. #** | **Interviewer question** | **Participant statements** | **Participant** |
| --- | --- | --- | --- |
| 1 | Und haben Sie irgendwie anderswo, anderswie nach Alternativen gesucht oder in irgendeiner Art und Weise sich versucht dazu zu informieren, was Sie tun können?  [And have you maybe looked elsewhere or in other ways for alternatives or in any way tried to educate yourself on what you could do?] | *Ja, ich hab ähm natürlich ein bisschen gelesen und hab dann überlegt mhm…was sind so Faktoren, die das positiv beeinflussen können ähm..wie die Ernährung, die Bewegung, genügend Schlaf ähm..Entspannung*, also so Techniken der Entspannung und *ja, eine Zeit lang hab ich meditiert auch.* *Das hat auch einen guten Effekt.* *Verzicht auf scharfe Speisen hat einen guten Effekt.* *Das hab ich jetzt auch mit der Studie nochmal gesehen*, weil...ich greif schon wieder vor (lacht), genau, ähm *diese Studie mich dazu animiert hat, das tatsächlich doch mal genau zu beobachten. So genau hab ich das vorher gar nicht beobachtet.*  [*Of course, I did -ehm- a bit of reading and then thought -mhm – what are some factors that could influence it [the hot flushes] positively, like diet, sports, enough sleep, relaxation,* well like relaxation techniques, *and yes, for a while I also meditated. That also has a positive effect. Abstaining from spicy foods works good too. I recognized that better with this study,* because now I’m jumping the gun again (laughs), right, ehm *the study has actually motivated me to observe it more closely. I haven’t observed it that closely.*] | Marlene |
| 2 |  | So …. *Und so hab ich dann eben halt versucht dann am Tag, also vormittags das alles so ein bisschen langsa – entspannter anzugehen und mich selber nicht zu stressen und dann wurde das auch so ein bisschen, hatte ich das Gefühl,* so ein bisschen besser, *also .. ich konnte es so ein bisschen regulieren.*  [*Then during the day I just tried to take it slo- more relaxed, and to not stress myself out so much and that’s when it’s gotten a little bit better – so I felt –* a bit better, *well, I could regulate it a little.*] | Andrea |
| **3** |  | *Und das war auch ja deutlich* sonst, also das hat sich da dann natürlich auch nochmal raus kristallisiert, *dass jetzt..auch während der Einnahmezeit,* also wenn ich das so angeguckt hab und ich denk das werden Sie ja wahrscheinlich au..., *also hatte ich den Eindruck, dass zum Beispiel die Wochenenden ähm...dass ich da weniger Hitzewallungen hatte als unter der Woche.*  [*That became pretty clear that,* well that became apparent of course *also during the intake,* well when I did look at it and I think you probably did to[o]… *I was under the impression that on the weekend for example, I had fewer hot flushes than during the week.*] | Ingrid |

**Cluster D, Theme (9) Control over symptoms**

| **Qt. #** | **Interviewer question** | **Participant statements** | **Participant** |
| --- | --- | --- | --- |
| 1 |  | Das ist so wie jemand sich an die *Zigarette klammert*, weil er glaubt dadurch ruhiger zu werden. Und so hab ich mich eben an dieses Placebo geklammert.  [It’s like someone *clinging onto cigarettes* because he believes he can calm down through that. And I’ve just clanged onto this placebo.] | Edith |
| 2 |  | *Hormone und so weiter, das ist ja etwas konkretes dann halt auch schon und ehm..es machts einem schon leichter wenn man das Gefühl hat okay, man..man versucht etwas, es gibt aber nur keine Nebenwirkungen*  [*Hormones etc. are also something that’s concrete, and -ehm – it does make it easier for you when you have the feeling that, ok- here’s something you can try, only that there are no side effects*] | Meike |
| 3 |  | Also ne, die Hitzewallungen sind ja in ihrem Ausmaße halt deutlich weniger geworden und *ich glaube der Schritt hin zur Annahme ist dadurch einfacher geworden, so*. Also wenns krass geblieben wäre, wär ich wahrscheinlich nicht, weiß ich nicht..ehm..*Ich hatte nicht mehr so schlimme, ganz schlimme Schlafstörungen und so weiter und war dadurch vielleicht auch ein bisschen klarer im Kopf, so, um mir darüber ein bisschen mehr Gedanken machen zu können*.  [Well, the hot flushes have become significantly less and *I think it has just gotten easier to accept it, yeah.* Well if they remained that extreme, I probably wouldn’t, I don’t know..ehm.. *I didn’t have such severe – those super severe sleeping problems etc. and because of that I was probably a bit clearer in my head I could also reflect a bit more about it.*] | Meike |
| 4 | Sie hatten das ja schon öfter, das Annehmen, angesprochen. Können Sie grob sagen zu welchem Zeitpunkt das für Sie anfing, dass das besser ging?  [You've already mentioned this, the acceptance, several times. Can you roughly say at what point it has started to become easier for you?] | Also, ganz klar tatsächlich mit dem Beginn der Studie irgendwie und dem, *der Erfahrung irgendwie, dass ehm..dass also eine gewisse Beeinflussung irgendwie da sein kann und es mir dadurch etwas einfacher gemacht wird.*  [Well, very clearly actually with the beginning of the study somehow and with the, *the experience I guess, that- ehm- a certain influence is in some ways attainable and that made it easier for me*.] | Meike |
| 5 |  | *Genau und das ist ähm halt was, was ich da auch sehr (stark betont) Positives mitnehme, also das stärkt ja auch die…die eigenen Kräfte und das eigene Selbstverständnis wieder, ne? Dass man da so viel in der Hand hat.*  [*And that’s exactly that one thing, that for me is a VERY positive takeaway – I mean it strengthens your …your own powers and self-image, right? That a lot is in your own hands.*] | Marlene |
| 6 | Können Sie da nochmal ein bisschen genauer den Unterschied erläutern, also sozusagen wie war es vorher, wie war es dann und woran haben Sie das ausgemacht?  [Can you explain the difference a bit more precisely, so to speak, how was it before, how was it then, and how could you tell?] | *Und als ich die Tabletten dann die vier Wochen nicht mehr genommen hatte war es dann irgendwie halt so wieder, als wenn das so ein bisschen da noch wieder war, so ein bisschen erhöhte (…) aber dann hab ich mir so, selbst wieder so, ach jetzt entspann dich mal. Fang nicht wieder an dich jetzt irgendwie so reinzusteigern, irgendwie halt. Und dann ging es auch dann nachher wieder weiter runter. Also das war ganz gut.*  [*And then after I haven’t taken the tablets for those four weeks, I was a bit under the impression that they came back and increased a bit. […] But then I told myself, oh come on, just relax a bit. Don’t get worked up about it all over again, something like that. And then it’s gotten back down again. Well, that was pretty good.*] | Andrea |

**Cluster D, Theme (10) Agency**

| **Qt. #** | **Interviewer question** | **Participant statements** | **Participant** |
| --- | --- | --- | --- |
| 1 |  | *Man hat sich allein schon hierher bewegt, irgendwie, mal real gesprochen, aber indem..man ist ins tun gekommen,* so, übers Gespräch, über die Einnahme von Placebo und so weiter und ehm..*das macht auch was mit einem, glaub ich.*  [*Just by getting things rolling, coming here- literally, getting proactive,* like that, through the conversation, through taking the placebo and so on and ehm *I think that does something to you.*] | Meike |
| 2 |  | *Ich fand das sehr schön* wie Frau Pan das so gemacht hat, *die Gesprächsführung, die Fragen, die sie so gestellt hat, dieses Selbstreflektive, was dann eben auch so an die Oberfläche gekitzelt wurde, das fand ich gut. Und das hat die Wahrnehmung natürlich auch nochmal so in diese Richtung gelenkt, also...versachlicht und eine größere Akzeptanz halt.*  [*I really thought it was really nice* how Ms Pan did it, *the way she held the conversation, the questions she asked- ones where you had to really reflect upon, those that went beyond the surface, I liked that. And that of course also directed the perception towards a more … well objective level and helps with the acceptance.*] | Marlene |
| 3 |  | Und ich finde, und bei mir ist es halt wichtig, das hab ich hier auch schon gesagt, aber es ist wichtig: *Ich MUSS darüber reden und hier konnte ich auch darüber reden.*  [And I think, and that’s something important to me, I’ve also already said that, but it is important: *I HAVE to talk about things and here I could talk about it.*] | Eva |
| 4 |  | Na, das ist...ich weiß nicht wie ich nochmal „*innere Haltung“* anders beschreiben soll, also ähm... Ja, meine Einstellung dazu, *dass ich der Meinung bin ich tu mir damit was Gutes, ich tu was für mich*..  [Well, that is… I don’t know how I should describe “*inner attitude*” differently, well ehm yes my attitude towards it, *that I think I am being kind to myself, I am doing something for me*.] | Ingrid |
| 5 | Wie war das für Sie an der Studie teilzunehmen? [What was it like for you to participate in the study?] | […] *Ich hab es wirklich sehr sehr sehr (Wiederholung) gerne gemacht,* die Betonung auf sehr, ich hab mich wirklich gefreut..ja, weil es etwas war, was ich für mich gemacht habe, ne? *Das ist auch so, dieser eine Aspekt, da kann mir keiner reinreden, das ist meine Entscheidung gewesen* und..egal wer was dagegen sagt oder ob (unverständlich), aber *es ist für mich gewesen*. *Das war mir sehr wichtig. Ein erster Schritt auch das zu lernen, genau.*  [*I was really very very very happy [to participate]* The emphasis on very, I was really pleased…yes, because it was something that I did for myself, your know? *It’s like, there is this one thing that no one can meddle with, it was my decision,* and…doesn’t matter who says something against it or whether (inaudible), but *I did it for myself. That was very important to me. A first step towards also learning this, yeah*] | Edith |
