# Supplementary material for: “Let’s see what happens:”—Women’s experiences of open-label placebo treatment for menopausal hot flushes in a randomized controlled trial
Source: PLoS One. 2022 Nov 4;17(11):e0276499. doi: 10.1371/journal.pone.0276499 (PMC9635716; doi:10.1371/journal.pone.0276499)
Supplement: Attachment — Submitted filename: OLP qualitative study - Response to reviewers - 220621.docx [file pone.0276499.s004.docx]

Dear Dr Nascimento,

Dear Dr Locher,

Thank you for taking the time to read our manuscript. You have raised some excellent points, and we were happy to make the respective changes.

We would appreciate it if you could have a look at the revised manuscript. Please feel free to raise any further points where applicable. We have included our answers to your comments in a different font and in blue. Please note that all page and line references refer to the marked-up copy.

Once again, thank you for your time and contribution to improving our work!

With my kind regards and on behalf of all authors

Yiqi

Reviewer #1:

The manuscript is very well written, appears thoroughly prepared and contains crucial contribution to the field of OLP and placebo research. The methods are precisely described and the results are very interesting.

Thank you for these kind words. We appreciate that you enjoyed the reading.

I have some remarks:
- There are references lacking in the introduction (e.g., 34, 37, ff).

We have added references to these statements (page 2, lines 36, 39, 40, 41).

- Line 50: I would not write „recently“. It is not so recent anymore.

Excellent point. We have removed the word “recently” (page 3, line 53).

- You might think about omitting the p values of your study in the intro for better readability

We have removed the statistical information in the introduction (page 3, line 60 and page 4, lines 77ff) and agree that it became more readable.

- It would be helpful to have the verbal script of the OLP treatment rationale in the appendix or a link

We have included the script of the placebo rationale in Supplementary File 1 (pages 35 & 36 in the manuscript, reference to S1 on page 5, line 95).

- Please provide also the a priori defined questions for the two blocks. It would be interesting to know, how many of these questions were asked in the interviews.

You are right that the original manuscript did not provide enough information about which questions were asked. We have now included all the guiding questions in Table 1 (page 7) and added further information in the text (page 7, lines 138ff).

- Further, original comments in German might be added to the supplement.

You may now find the original citations in Supplement 3.

- You could omit to mention the RCT results again in the methods section.

We removed the redundant information from the methods section (page 5, lines 97ff).

- Regarding the potential bias for only interviewing women who showed improvement: You may make this transparent already in the abstract: “women who benefited…”

You make an excellent point and we have changed various sentences according to your suggestion (Abstract: page 2, line 25 & 34; Introduction: page 3, line 66; Discussion: page 22, line 461 and page 28, line 591)

- Table 1 might be adapted: Substitute the changed names with the N of participants who mentioned the theme. This would give a better overview and enable more focus on the themes. The themes could also be highlighted (e.g., in bold) and the letters A-D in a first, separate column.

We agree that Table 1 looks cluttered, so we removed the names. We have also happily adopted your suggestions to better structure the table. However, we wondered whether adding the N of the participants might make some themes appear more “representative” than others, which is not a claim we are trying to make with our qualitative data. Therefore, we simply removed the third column.

- Sociodemographic data of the qualitative sample is missing. Please add descriptive information.

We have added sociodemographic information relevant to the interpretation of the data in Table 3.

- Did you correct the verbatim grammatically? The statements seem very structured.

We first had our citations translated through software and then made manual adjustments afterward. It seems that some of the filler words were indeed lost through the software. We looked through all the citations and re-added the filler words.

Small correction suggestions:
. line 52 e.g., 13 RCTs

Thank you for pointing this out. We have changed RCT into RCTs (page 3, line 55).

. , at least not part of the study

As this is already the wording (page 14, line 277), we are afraid we are not sure what you are suggesting. We might be missing something and would appreciate if you could provide us with more information.

. line 422 placebos being “better than nothing” treatments

We have included this term in brackets and added two citations (page 24, line 507)

Reviewer #2: Abstract

line 21: It is not true that no study investigated patients’ experiences undergoing treatment. There are now at least two studies who did so:
Bernstein MH, Magill M, Weiss A-P, et al. Are conditioned open placebos feasible as an adjunctive treatment to opioids? results from a single-group dose-extender pilot study with acute pain patients. Psychother Psychosom 2019;88:380–2.
Locher, C., Buergler, S., Nascimento, A. F., Kost, L., Blease, C., & Gaab, J. (2021). Lay perspectives of the open-label placebo rationale: a qualitative study of participants in an experimental trial. BMJ open, 11(8), e053346.

It’s very kind of you to provide these citations. We also found two more studies, Haas et al. 2022 and Sandler et al. 2008, and included relevant findings on:

- Page 15, lines 301ff.
- Page 23, lines 474f.
- Page 24, lines 492ff.
- Page 24, lines 497f.
- Page 26, lines 538ff.

Furthermore, we deleted the sentence “no study has yet assessed…” (page 3, line 61).

line 28: the summary of the abstract seems very optimistic, considering that many patients had low expectations about the placebo treatment. I would urge that the authors list pro and cons of the approach

You are right that our abstract only included “positive” aspects of the OLP approach, which might bias the reader into thinking that OLP is particularly useful. The relatively positive depiction arises through our sample – as we have only included women who benefitted from the treatment. You and Reviewer #1 helped us see that this information is crucial, which is why we have modified the abstract (page 2, lines 25f & 34) and other sections in the manuscript accordingly (Introduction: page 3, line 66; Discussion: page 22, line 461 and page 28, line 591).

As for listing the pros and cons – we absolutely agree that it is imperative to better understand what participants think about the OLP approach. Therefore, we had already asked participants whether they considered OLP use legitimate in clinical practice in the RCT. (<https://static-content.springer.com/esm/art%3A10.1038%2Fs41598-020-77255-z/MediaObjects/41598_2020_77255_MOESM2_ESM.pdf>)

Hence, in this qualitative study, our focus was a different one. Our objective was not so much to assess their opinions on OLP, placebos, or the placebo effect. Instead, we aimed at understanding the experiences of menopausal women taking OLP and interpreting these experiences in light of their symptom history and attitudes towards their symptoms. We hope that our recent modifications reflect our study goal more clearly (page 3, lines 65ff., page 27, lines 568f.)

Introduction

line 38: the authors state that recent research into the neurobiological underpinnings of placebo effects have been conducted. I would prefer a list of references after this sentence.

We have added these references (page 2, line 41).

line 58: again, it is not true that no study has yet assessed participants’ experiences taking OLP (Bernstein et al.; Locher et al; see abstract comment). I would recommend that the authors briefly discuss the findings of these qualitative studies and outline how their approached differed from them.

We have included a paragraph on the other qualitative studies (page 26, lines 546ff.)

Methods

lines 76: the original RCT included 100 women suffering from hot flushes. The nested qualitative study only conducted interviews with a total of 8 patients. This is a very small sample size, also for a qualitative approach.

line 99: I am also very worried that the authors only included patients with an experience of relief in the RCT (i.e., a score of 5 or higher on the overall improvement scale [range from 1-7] was mandatory in order to take part in the qualitative study). I think it would have been much more representative if participants with and without symptom relief would have been eligible. This would allow researchers to learn from both subgroups and to have an in-depth insight about chances and hurdles of the OLP approach.

Your feedback helped us understand that we didn’t sufficiently describe our decision-making considering sample size and sample.

Our decisions are founded on the method used (IPA) and our study goal.

On page 6, lines 118ff., we have now added a more detailed explanation: “We opted for this selective focus and small sample size to (a) ensure a feasible analysis given the rich data generated and (b) because a homogeneous and small, purposively chosen sample is considered appropriate when applying the IPA method [20]. Our sample size aligns with the standard of IPA studies of including five to ten participants [19, 23, 24]. “

We have also more clearly outlined our study goal starting page 3, lines 65ff: “In this study, we investigated the experiences of menopausal women who underwent and benefitted from OLP treatment for hot flushes. Our primary interest was exploring each participant's individual treatment experiences in light of their symptom history and attitudes towards their symptoms.”

The sentence “Such understanding may help us access the acceptability, practicality, and methodology of placebo administration” (lines 82ff.) hints at the potential of being representative (which we cannot and are not aiming at achieving), is misleading, and has been removed.

Similarly, we have removed the sentence “Our findings enable us to gather a first understanding of participants’ experiences with OLP and to contextualize the findings from OLP RCTs” from the limitations (page 27, line 569) and the sentence “…these factors […] may be pertinent to biomedical and CAM treatments as well” from the conclusion (page 28, lines 597ff).

We agree that our approach had the downside that we could not compare participants with and without symptom relief. We have acknowledged this limitation (page 27, lines 567ff), and added a relevant finding from the Haas et al. 2022 study, which did compare non-responders to responders.

line 11: the authors designed two blocks for the unstructured interviews, whereby the first block focused on symptom history. The authors state that the second block, which concentrated on placebos, was the focus of the current analysis. Why did the authors create two blocks and then only focused on one of them? I would advise that both blocks are considered equally important for the results, especially with the claim that these two blocks interact (line 116).

Excellent point. We can see how naming the blocks without describing them in detail is confusing to the reader.

In a nutshell, Block 1 established the context for Block 2. Block 1 helped the interviewer understand the problem/initial situation of the woman and enabled her to know what issue to explore in-depth and to ask good questions specific to each woman’s situation.

We made the following changes:

- Added the guiding questions (Q1-Q3 refer to Block 1, Q4-Q7 refer to Block 2)
- Clearly outlined what is meant with “focusing on Block 2 for the analysis” in the Analysis section (page 8, lines 161ff.) and adjusted the Data collection section (page 6, lines 138ff).
- Added information on the aim of the IPA method to introduce the concept of context (“IPA accentuates that the researcher should strive to understand the participant’s experiences in context of the participant’s realities.”, page 5, lines 105ff.).

Results

Table 1: all themes listed in table 1 are positive attitudes towards the OLP approach. This is surprising since the authors also state that “almost all women reported that they had little expectations”. I would urge that the authors build an independent category for this statement and also list it in table 1. A statement like “I approached it without too much thinking. It also wouldn’t seem logical to me, that I’d come out all healed” was listed under the main category “openness and hope”. This is not at all intuitive to me.

Thank you for pointing this out.

We have now more clearly differentiated between expectations towards the OLP treatment and attitudes towards placebos in general. We define expectations in line with Rief & Petrie as “specific cognitions about the likelihood of future events” (added in the Discussion, page 23, lines 471ff). These expectations were rather low.

Nonetheless, our interviewees had an overall positive attitude towards placebos. We presume that this is because the RCT was already advertised as a placebo study (this information is now added on page 23, lines 468f.). Those who did call in for the RCT declined participation once they knew it was a placebo study (n=9) (<https://www.nature.com/articles/s41598-020-77255-z/figures/1>). Therefore, the RCT acted as a filter for individuals who were highly skeptical towards placebos.

We agree that the statement you mentioned is indeed ambiguous. We have extended the statement and added further information (page 12, lines 214ff.).

We had debated creating a stand-alone category for expectations. However, we decided against it in the end as the hopes and expectations derive from the women's open attitude. They are open to uncertainty (hence low expectations - something good may or may not happen), but they are hopeful enough to make an attempt.

line 252: in one of the citations, linguistic reinforcement was underlined by the authors. I would recommend to do this consistently throughout all citations (especially because the authors did not apply a content/thematic analysis that only concentrates on the content of the quotations but rather more a phenomenological approach that is interested in the lived experiences of participants)

This is an excellent idea that we have gladly implemented on page 11, lines 201f, page 12, lines 222f, and page 15, lines 301ff.

Discussion

line 392: the authors state that participants were hopeful, curious, fascinated, and at ease. Again, I think that low expectations should also be discussed. The finding that all women had a relatively positive view of placebos when entering the trial could be associated with the fact that they all experienced symptom relief (i.e., which could have been the cause of a positive attitude).

We extended the section by discussing expectations further (page 23, lines 470ff and page 28, lines 594f.). As mentioned, we believe that our quantitative study only attracted individuals open to placebos in the first place. Almost all our participants had taken CAM before, indicating their endorsement of holistic treatments and the mind-body connection with which they also associate with the placebo effect. As study participation meant committing to attending four on-site appointments, completing a symptom diary and (potentially) taking pills daily, only women who hope to gain from the treatment were likely to participate.
