# Supplementary material for: “Let’s see what happens:”—Women’s experiences of open-label placebo treatment for menopausal hot flushes in a randomized controlled trial
Source: PLoS One. 2022 Nov 4;17(11):e0276499. doi: 10.1371/journal.pone.0276499 (PMC9635716; doi:10.1371/journal.pone.0276499)
Supplement: Attachment — Submitted filename: OLP qualitative study - Response to reviewers R2- 220919.docx [file pone.0276499.s005.docx]

Dear reviewers,

Thank you for taking the time to review our manuscript!

We were happy to incorporate your feedback in the revised version. Please note that all line references refer to the marked-up version.

With my kind regards and on behalf of all authors,

Yiqi Pan

-----

Reviewer #1:

I thank the authors for considering my suggestions and addressing my concerns.
The authors may consider excluding a remaining opening bracket in line 94 and change: „Was“ instead of „What“ in Table 1 in Q2.

Wonderful, thank you! We have changed the typo in Table 1 and removed the open bracket in (what is now) line 72.

------

Reviewer #3:

TITLE: This particular study where 8 women where interviewed after administering a placebo is supposed to be a randomized control trial however, it is not clear when the randomization was done hence the title seems to contradict the study design.

Thank you for raising this issue. We have added a specification in line 60 (italic): “In this study, we investigated the experiences of menopausal women who underwent and benefitted from OLP treatment for hot flushes *that was administered as part of an RCT.”* We hope this specification early in the manuscript will guide the reader’s understanding of the study’s design.

Moreover, we have added that the eight women we’ve approached and included in this qualitative study are the first eight women who have improved in the quantitative study (page 5, line 107f.)

I9NTRODUCTION: The 1st sentence in page 4 is not clear, did the placebos significantly reduce or increase menopause related quality of life?

Thank you for pointing this out. We corrected the sentence (page 4, line 70).

METHODS: The 1st sentence which states states that the study is the second part of a design is not clear and this is not referenced?

In line 77, the quantitative study referred to is not referenced and no information or synopsis is given for better understanding .

Excellent point – we have now added the references for the study protocol and the results of the RCT. To clarify the “explanatory sequential mixed-methods design” (line 75) that we’ve used, we added a sentence on page 4, line 75ff (“Details on the design of the preceding quantitative study, i.e., the RCT, are provided in the study protocol [17] and the RCT results publication [16].”).

A very brief summary of the RCT’s results is provided in the introduction (lines 69f.), and a synopsis of the RCT’s design is given in the “Design” paragraph (lines 77 – 89).

In line 78, what do the authors mean by -- moderate subjective burden? In line 79, what do the authors mean by --- one week run in to access base line symptoms and what are these symptoms?

We agree that these terms are unclear and have done some rewrites (lines 77 – 81): “In brief, 100 women in peri- or post-menopause who had at least five hot flushes per day and were moderately or severely burdened by their symptoms were randomized to receive four weeks of OLP or no treatment. Before the randomization, all participants protocoled their hot flushes for a week (baseline assessment).”

In line 88, COREQ should 1st be written in full.

We’ve added the name in full (line 91).

Line 89, which protocol are the authors referring to and how is it related to this study?

We refer to the study protocol and have added the word “study” (line 93) for further clarity.
